# Complaints to the veterinary disciplinary board related to the euthanasia of animals

**DOI:** 10.3389/fvets.2024.1480106

**Published:** 2024-12-16

**Authors:** Charlotte Berg, Hannah Vickers

**Affiliations:** Department of Applied Animal Science and Welfare, Swedish University of Agricultural Sciences, Skara, Sweden

**Keywords:** animal welfare, companion animals, complaints, euthanasia, One Welfare, veterinary clinic, VDB, VMB

## Abstract

This article presents an analysis of the formal complaints to the Swedish Veterinary Disciplinary Board (VDB) during the years 2018–2022 related to euthanasia of animals, which are highly relevant to the One Welfare approach. The aim was to examine whether the complaints were justified or not, according to the disciplinary investigations carried out, and whether animals had been exposed to unnecessary suffering during these interventions. The reasons for the complaints were investigated and categorized. The results showed that incorrect treatment or handling of the animal was the most common reason for reporting a veterinarian to the VDB, while the main underlying cause, based on the qualitative analysis, was found to be communication barriers. However, the number of complaints leading to disciplinary outcomes (admonitions or warnings) was very low. The vast majority (45 out of 47) of complaints relating to euthanasia were therefore not legally justified as per the VDB decision, as the veterinary staff were found to have acted correctly from a veterinary and animal welfare perspective. Nevertheless, even unjustified complaints can be very stressful for the veterinarian and should be minimized.

## Introduction

In today's society, companion animals are important to many people and are often considered family members rather than possessions (1, 2), and the welfare of the animal will influence the welfare of the owner, and vice versa (3). When companion animals, also known as pets, become ill or injured and require medical care, their owners will turn to veterinary professionals for help. This includes advice and support at delicate or sensitive moments, such as euthanasia. Expectations are high for veterinary staff in these situations, as they need to be medically correct and able to deal with emotional clients. Emotional situations can easily arise at the time of euthanasia, as many clients will grieve for their pet in a manner similar to the loss of a close friend or relative (4). From an animal welfare perspective, euthanasia may be the intervention of choice when an animal is terminally ill or when the prognosis for recovery is poor despite treatment efforts. Euthanasia is also sometimes used when treatment is simply too expensive for the client, or in the case of dangerous dogs that have, for instance, repeatedly and violently attacked people or other dogs. From the veterinarian's point of view, being able to offer euthanasia can be a relief, as it may be the best option to end or prevent severe animal suffering. In such a case, it can be very frustrating and stressful for the veterinarian if the client refuses to consent to euthanasia (5). On the other hand, a situation where the client requests the euthanasia of a healthy pet and refuses all efforts to relocate the animal can also be stressful and frustrating for the veterinarian (5).

Society places a high value on access to well-trained veterinary professionals, including veterinary technicians. These professions are of paramount importance in maintaining good animal health and good animal welfare (6), since good veterinary care is often a prerequisite for preventing unnecessary suffering. Furthermore, it is also closely linked to human welfare, as—in line with the principles of One Health and One Welfare (3)—the welfare of the owner of a companion animal is closely linked to the welfare of the animal. Moreover, the veterinarian's wellbeing may be compromised in situations where animals are suffering. Sometimes, however, the animal health care system does not deliver according to standards and expectations. This can have consequences, partly in terms of dissatisfied clients, but also potentially in terms of animal suffering. In cases where the owner is dissatisfied, he or she can make a formal complaint to the Veterinary Disciplinary Board (VDB), sometimes known as the Veterinary Medical Board (VMB) (1), in the relevant country to have the actions of the veterinarian or other certified animal health care personnel evaluated from a legal and medical perspective.

A VDB can be found in many countries around the world, although the organization and procedures of such bodies can vary considerably between countries, and are sometimes established at a regional state level (1, 7). In some cases, the VDB is run by the government, in others by a branch of the veterinary association. In some cases, it is linked to the professional body that issues licenses to practice, in others it is not. The VDB may only cover licensed veterinarians, but in some countries it also covers other licensed animal health professionals, such as veterinary technicians, certified farriers, and dentists working at veterinary clinics. In this paper, the term “veterinarian” is used because no other animal health care personnel are responsible for euthanasia of small animals, and therefore not in charge in the cases investigated here.

The Swedish VDB is a competent authority whose work is guided by a number of laws, such as the Animal Health Care Act (Lag om verksamhet inom djurens hälso- och sjukvård) (8) and the Animal Welfare Act (9). Complaints can be made to the VDB by animal owners/keepers and the competent authorities if they believe that a veterinarian has been involved in malpractice. This includes deficiencies in record keeping and carelessness in the writing of veterinary health certificates. Depending on the nature and seriousness of the malpractice, an admonition or a warning may be issued (proposal 1995/94) (10). In the case of very serious or repeated malpractice or misconduct, measures such as a 3-year probationary period or withdrawal of the license to practice may be applied (11). Other types of sanctions or disciplinary measures that may be applied in other countries or states are mandatory ethics training, community service, continuing education or fines (7). However, these are not included in the Swedish VDB system.

In this paper, the terms “owner” and “client” are used to describe a person who owns, keeps or is otherwise responsible for the care of a pet, regardless of formal ownership. It is well-known that complaints made to a VDB are not always well-founded from a veterinary perspective, i.e., malpractice has not necessarily occurred each time a complaint is made (7); this is for the VDB to investigate. As pet owners are often emotionally involved when bringing their pet to the veterinary clinic, they may be less receptive to important information (12) and misunderstandings may therefore occur.

The aim of this study was to analyse the complaints to the Swedish VDB, to find out the frequency of euthanasia-related complaints, what species they concerned, what the reasons for the complaints were, what the outcome of the complaint is in terms of decisions made by the VDB, and whether any types of animal welfare consequences of such malpractice were identified.

## Materials and methods

During the period 2018–2022, a total of ~1,000 complaints were submitted to the Swedish VDB, which corresponds to ~200 cases per year. For this study, copies of all cases of complaints related to euthanasia made to the Swedish VDB during this period were requested, including replies and copies of records from the veterinarians involved, as well as the decision and motivation written by the board. In total, there were 53 cases involving complaints related to euthanasia. Six of these were excluded as they did not meet the inclusion criteria (e.g., the complaint did not relate to the act of euthanasia, or the person making the complaint was not the owner of the animal or did not represent the authorities and was therefore not authorized to file a complaint). The remaining 47 cases concerned only companion animals, of which dogs were the most common (55%), followed by cats (38%), horses (4%, i.e., two cases) and one hamster.

The complaints were classified according to the reason for the complaint as stated by the client. The documents were also analysed for information on animal welfare issues, counterclaims and details of the underlying reasons for the complaint. Descriptive methods were used to illustrate the numeric data, and a thematic analysis approach was used for the qualitative analysis of the data.

## Results

### Reasons given for complaints

Two main reasons for filing a complaint in relation to euthanasia were identified, based on the clients' own classification. The most common reason for reporting a veterinarian to the VDB was incorrect treatment or handling of the animal, i.e., improper or negligent professional behaviour, commonly referred to as malpractice. This finding is consistent with previous findings by Babcock et al. (25), although the exact definitions and classifications may not be entirely the same. In our study, the majority of cases were classified as incorrect treatment, which means that the veterinarian has chosen a treatment method that is not considered correct or optimal for treating the disease or injury in question (11). An example of this is when the animal was euthanised when other types of action or treatment would have been preferred, or when the client felt that the handling prior to euthanasia was not correct. Another, less frequent, classification was mistakes in the exercise of the veterinary profession, e.g., applying the (correctly) chosen method of treatment in an incorrect way (11).

### Notification to the authorities

In addition to analysing the reason for the complaint as stated in the documentation, it was also noted whether the veterinarian had sent an animal welfare notification to the competent authorities regarding the status and condition of the animal. According to national legislation, animal health professionals are obliged to notify the authorities if they have reason to believe that the client has violated animal welfare legislation (9). This was investigated since such a notification may result in the client retaliating by filing an (unfounded) complaint toward the veterinarian. This was found to be the case in 9% of the complaints analysed.

### Clients' expectations

A qualitative analysis was carried out in order to investigate what the clients' expectations were in relation to the actual euthanasia, and what aspects of the euthanasia act the clients felt did not meet these expectations.

Our analysis showed that in the majority of cases (57%), the euthanasia process failed to meet the expectations, and was therefore the main reason for the complaints. For example, one client stated that the process was not as calm and peaceful as they had expected and this was the reason for their complaint. We did not assess whether the clients' expectations were reasonable or not.

### Outcome of complaints

Two (4%) of the complaints related to euthanasia filed in 2018–2022 resulted in a negative outcome for the veterinarian, involving a cat and a dog (Table 1). Both were cases of incorrect treatment, where both veterinarians received an admonition, the milder form of reprimand that the VDB can issue. In one case, the veterinarian performed an intracardiac injection of pentobarbital as the euthanasia method, which is acceptable only if the animal is unconscious, according to the Swedish animal welfare legislation; however, in this case, it was not. Therefore, the veterinarian had euthanised the animal in an incorrect manner and not complied with animal welfare legislation, in a way that may have caused distress to the animal. In the other case, the VDB was of the opinion that the veterinarian did not carry out a complete diagnostic procedure of the animal and that the veterinarian should have recommended a medical treatment prior to presenting the other treatment options, including the option of euthanasia. Therefore, the veterinarian was found to have made an incorrect recommendation about the possible treatment options for the animal. This means that this complaint was not about the way in which the euthanasia was actually carried out, but about the way in which the decision to euthanise was taken. As death *per se* is not considered an animal welfare problem (once an animal is dead it cannot suffer), the problem here was rather about not giving the animal the opportunity to have a longer life.

**Table 1:** The number of and the outcome of complaints to the Swedish VDB related to euthanasia of animals 2018–2022.

**Year**	**Number of complaints**	**Outcomes: admonition (A) or warning (W)**
2018	6	2 (A)
2019	16	0
2020	7	0
2021	12	0
2022	6	0
Total	47	2

### Qualitative analysis of the reasons for the complaints

The thematic analysis of the tours identified two distinct themes and three subthemes (Table 2). The themes were “Interaction between the client and the veterinarian” and “The Client's feelings”, with “Interaction between the client and the veterinarian” being the most prevalent in the reviewed documents (Table 3). In the following section, the subthemes of these themes are presented.

**Table 2:** Summarised results from the thematic analysis of the complaints to the Swedish VDB related to euthanasia, in which two main themes and three subthemes were created.

**Theme**	**Subtheme**	**Code**
Interaction between the client and the veterinarian	Communication	- Lack of instructions - Communication barriers - Veterinary time constraints - Language barriers - Empathy during euthanasia
	Client's attitude	- Verbal attacks - Distrust toward the veterinarian - Distrust toward the clinic
The client's feelings	Emotional bond to the animal	- Reluctance regarding euthanasia - Knowledge about animal behaviour - Incomprehension

**Table 3:** Number of complaints to the Swedish VDB related to euthanasia in each theme and subtheme generated from the thematic analysis.

**Year**	**Theme**	**Subtheme**	**Number of complaints**
2018	Interaction between the client and the veterinarian	Communication	2
		Client's attitude	2
	The client's feelings	Emotional bond to the animal	2
2019	Interaction between the client and the veterinarian	Communication	9
		Client's attitude	2
	The client's feelings	Emotional bond to the animal	5
2020	Interaction between the client and the veterinarian	Communication	3
		Client's attitude	1
	The client's feelings	Emotional bond to the animal	3
2021	Interaction between the client and the veterinarian	Communication	9
		Client's attitude	3
	The client's feelings	Emotional bond to the animal	0
2022	Interaction between the client and the veterinarian	Communication	2
		Client's attitude	1
	The client's feelings	Emotional bond to the animal	3
Total for all years			47

#### Communication

The subtheme “communication” covered a relatively large number of codes and communication problems were found to be a significant cause of many complaints (Table 3). It was clear from the documents that there was a lack of information given to owners about how euthanasia would be carried out and what to expect. For example, in six cases, clients stated that the veterinarian did not explain the procedure beforehand, or that the euthanasia was carried out very rapidly and they did not have time to say a proper goodbye to their pet, something which occurred in two cases.

In three cases it is also clear that the risk of complications associated with euthanasia was not explained—or understood—to the clients beforehand. This led to misunderstandings in one case, where the client misinterpreted a rare, but not unique excitation event in a dog as suffering and an act of cruelty, whereas the veterinarian had noted that some reflexes were seen during euthanasia, related to the depression of the brain after loss of consciousness.

There were also cases where there was a communication barrier between the veterinarian and the owner during the clinic visit. In five cases it was clear that the owner was unable to understand or comprehend what the veterinarian was saying about the animal's diseases, current condition and prognosis, and although they agreed to have the animal euthanised, the client later questioned why this was necessary in the first place. This includes one case where the veterinarian wrote in the records that a colleague was brought into the room to help explain the situation to the client, but this still did not make any difference.

One complaint stated that the veterinarian was stressed and did not allow enough time for the procedure, or at least that was the perception of the client. This was thought to have had a negative impact on the veterinarian's ability to communicate properly with the client. In one case, language barriers was also mentioned, where the veterinarian was not fluent or easy to understand in Swedish, potentially complicating communication and thereby negatively affecting the client's perception of the whole visit.

#### Client attitudes

As noted above, problems in communication between the veterinarian and the client were found to be a major reason for making a complaint. In addition, in nine cases (Table 3), it was evident that the attitude of the client may have contributed to the subsequent complaint. Six complaints contained negative statements about the veterinarian, condescension or suspicion, where the competence or actions of the veterinarian were questioned in an aggressive manner, such as accusing the veterinarian of withholding information or lying.

#### Emotional attachment to the animal

The strong emotional bond between owner and pet is reflected in 13 of the complaints (Table 3). In our study, it was clear that in seven cases, the reason for a complaint was that the clients felt that they had been persuaded to have the animal euthanised, and later complained when they regretted having agreed to the euthanasia in the first place.

There was also one example of how the emotional attachment can make the client upset if they feel like their animal is not being treated fairly, with their whole focus on their beloved animal. This particular case was mainly in response to staff requesting that the animal should wear a muzzle for their safety (as part of their routine or because of previous experience with a particular breed or the individual dog and its behaviour), which that client found intrusive or offensive. Such clients would not necessarily be willing to listen to an explanation saying that the veterinarian did not know the dog and wanted to be on the safe side.

## Discussion

The number of dogs and cats in Sweden is estimated to be around 1.1 and 1.4 million, respectively. During the period 2018–2022, dogs and cats completely dominate the number of complaints regarding euthanasia to the VDB, which might theoretically be interpreted as an indication of extensive malpractice and significant welfare problems in these species. For veterinarians and other animal health personnel, the complaints may also have significant welfare implications. Although justified complaints, i.e., complaints were there is solid ground for criticism according to the VDB decision, are important for the quality assurance of veterinary care in general, unjustified complaints can be detrimental to the veterinary practice. This is a concern in many countries, where dissatisfied clients unfairly use various legal tools against veterinarians (7). Additionally, also complaints where the VDB does not find a legitimate reason to criticise the veterinarian, may contribute to stress and possibly lower both self-esteem and job satisfaction among veterinarians. It is therefore important to investigate the reasons why clients make such complaints in order to prevent or minimise the number of unfounded complaints.

A very large proportion of complaints to the Swedish VDB do not result in an admonition or warning to the veterinarian(s) involved; only 0.7% of cases during 2019–2022 resulted in a warning and 14% in a milder admonition (13, 14). The present study also found low numbers of admonitions and/or warnings, and a majority of the complaints filed relating to euthanasia concerned companion animals, possibly because this is a particularly sensitive intervention.

### Reasons for the complaints

According to the files, incorrect treatment or handling of the animal was the most common reason for reporting a veterinarian to the VDB, followed by errors in the exercise of the veterinary profession. However, when analysing the complaints in detail, we found that this classification was partly misleading. Instead, the thematic analysis revealed that in many cases the underlying reason for the complaints was problems in the interaction between the client and the veterinarian, and the client's feelings, i.e., the client's challenges in dealing with their own feelings in such a difficult situation. There may be several explanations for the discrepancy in results between the initial quantitative analysis and the later qualitative analysis. One explanation may be that the client does not understand the remit of the VDB, which only relates to malpractice and other professional errors (11), or does not understand or is not familiar with the normal and expected process when an animal is to be euthanized. In such cases, the client may suspect mistreatment or incompetence on the part of the veterinarian, when the problem is more likely to be related to human interaction and communication, or even payment issues, which are outside the remit of the VDB. Lack of information from the veterinarian, or an inability to process the information provided in a difficult situation, can lead to some clients not understanding the procedures. In addition, clients who have observed slightly different procedures in previous cases, perhaps in other clinics, may react because the procedure is not what they expected based on previous experience.

Only a relatively small proportion (9%) of complaints were related to the veterinarian notifying the authorities about possible animal welfare violations. As we do not have access to information on the total number of such notifications or how frequent they are in relation to the cause of the complaint, we cannot draw any far-reaching conclusions from this. It could be hypothesised that such reports are less frequent in relation to euthanasia than for the average veterinary visit, if the veterinarian believes that a report is pointless once the animal is deceased. On the other hand, it could be hypothesised that an animal brought to the clinic for euthanasia is likely to be in poorer condition than the average patient presented for annual vaccination or neutering, and it is therefore a risk that the client may have waited too long before deciding on euthanasia. This was not investigated in this study, but may be of interest for further research.

### Interaction between clients and veterinarians

As mentioned, the interaction and communication between clients and veterinarians was found to be an important factor in why a formal complaint was made to the VDB regarding the euthanasia of companion animals. This is not surprising as visits to the veterinary clinic can be emotional events in general, and particularly in relation to critically ill animals or planned euthanasia. In such cases, it can be difficult for the client to process and understand the information provided by the veterinarian (12). In some cases, clients stated in their complaints that they did not receive information about the process of euthanasia or the risk of complications. In some complaints, clients stated that the veterinarian appeared to be under stress, time wise. For several years, a shortage of veterinarians has been reported in many European countries, including Sweden (15), and it is possible that a high workload for the clinic means that owners do not have enough time to ask questions before the actual euthanasia is carried out. Other studies have shown that some clients find it important for the veterinarian to be physically present at all stages of the euthanasia process (16). However, it is not known whether clients would be willing to pay for such an arrangement, where the veterinarian does not leave the room, e.g., between pre-medication and injection of the euthanasia drug.

Shortcomings in the interaction between the parties can also lead to mistrust of the veterinarian, other staff, or the clinic as a whole. One example of this is if the client notices discrepancies between the information given by different people in the clinic, or between the information given at the time of booking and what was actually experienced in practice. It is therefore important that the veterinarian is transparent regarding how the euthanasia will be carried out, possible complications that can occur, and the different stages of the process, even though such details can sometimes be difficult to discuss. Such information may need to be repeated on more than one occasion to reduce the risk of misunderstanding or negative attitudes toward the clinic. Furthermore, it has been shown that clients expect to be fully informed about the prognosis and possible treatment of a sick animal when visiting a veterinary clinic (17). It is therefore important to remember that in veterinary medicine, euthanasia is a possible, and sometimes encouraged, method of treatment to ensure that animals are not subjected to severe or prolonged suffering (18).

The decision about euthanasia is often a sensitive issue that many companion animal owners do not want to make, as the decision to euthanise a beloved pet or family can lead to feelings such as guilt and sadness (12), while at the same time they are aware that euthanasia may be the best choice for the animal in certain situations (2). It is therefore not surprising that clients will ask the veterinarian for advice when deciding about euthanasia and the procedures involved, as this may partially relieve the animal owner of the moral stress of such a situation (12). Leaving the decision to the veterinarian, on the other hand, may result in the client feeling out of control, persuaded or angry with the veterinarian who recommended euthanasia. By providing all available information, the veterinarian can give the client a good basis for making a decision without the risk of being perceived as “taking over” the process, thereby reducing the risk of conflict (12). However, the veterinarian must be aware that in many cases, as stated for example in the Swedish Animal Welfare Act, decisions should be based on what is best for the animal and on minimising unnecessary suffering. This means that the veterinarian must act by contacting the relevant authorities if a client's decision is completely against the animal's best interests.

### Clients' feelings

The death of a companion animal can cause deep sadness and grief for the owner if the human-animal bond is strong. Animals can provide affection, social interaction and security, thereby satisfying several human needs (19). The thematic analysis in this study showed that the emotional bond is important in influencing the client's behaviour and may explain why some are reluctant to make a decision about euthanasia. This bond may contribute to the client's inability to understand or accept the veterinarian's recommendation for euthanasia, even though it may have been the best solution for the animal.

The veterinarian cannot expect the owner to be familiar with the expected behaviour and reactions of the animal during the administration of euthanasia drugs, and lack of knowledge may be a factor in complaints to the VDB. If the owner perceives the euthanasia process to be painful or very distressing for the animal, this can be very traumatic for the owner if the emotional bond is strong. It is important for the veterinarian to be aware of the different possible expressions of human behaviour at the loss of a pet, where shock, denial, distrust, anger and alienation may be steps that the client goes through, and that these feelings may be channeled into frustration and anger and a desire to find someone to blame (20).

There are things that can be done to help bereaved pet owners, increase customer satisfaction and reduce the risk of complaints. Although it may seem cynical at first, helping the client can also help the veterinarian. For example, a short note of condolence can be sent, or a phone call can be made, to the client shortly after the euthanasia event (2). This may be a time-consuming task, but it may reduce the number of complaints, which are also time-consuming. Some veterinary hospitals and practices in North America have employed “veterinary social workers”, a social worker who specialises in helping both clients and veterinarians in their relationships with animals (21). Death and bereavement counseling is one area in which such a person can help, increasing the client's understanding of the animal's situation by calmly discussing the various treatment or non-treatment alternatives, emphasizing the animal's interest in avoiding severe pain and suffering, and reducing the risk of misunderstanding. Of course, the clinic will have to balance this preventative work and the reduced workload for the veterinarian against the potentially increased cost of employing this type of staff and the general cost of taking a pet to the clinic.

### Outcome of complaints

As shown above, the number of complaints against veterinarians related to the euthanasia of animals in Sweden in 2018–2022 was 47 in total, of which only two resulted in an admonition (and none in a warning or more severe intervention). It is thus unusual for a complaint to be considered well-founded from a veterinary perspective; in the vast majority of cases, the veterinarian acted correctly based on science and established medical practice. This supports our conclusion that communication and interaction problems between the client and the veterinarian, and the client's feelings, underlie many of the complaints. Only in one of the two cases resulting in a disciplinary outcome, animal welfare aspects may be included in the reasoning from the VDB, i.e., for 2% of the total complaints investigated in this study. From this, we can conclude that the number of complaints to the VDB related to euthanasia is not a useful indicator of animal welfare-related malpractice resulting in unnecessary suffering.

### Sustainability and societal perspectives

Veterinarians and others working in the field of animal health often have to make difficult decisions and trade-offs in their daily work in order to maintain a good level of animal welfare and to meet the demands for certain treatments expected by clients; two aspects that do not always coincide and can sometimes represent conflicting interests (5, 22). Euthanasia is just one of many ethical dilemmas that practicing veterinarians have to deal with, contributing to moral stress (23). Some clients have unreasonably high expectations of the outcome of treatment for disease or injury and will not be satisfied with what can be achieved, which can lead to stress and pressure for the veterinarian (24). Some veterinarians find it more difficult to satisfy clients with companion animals than farmers with production animals (24), and this may be one of the explanations for the lack of complaints related to euthanasia of livestock identified when scanning the material for this study. Another reason may be that the euthanasia of farm animals, if necessary, is often carried out by others than veterinarians, such as the farmer, farm workers, a knacker, or an experienced hunter in the neighborhood.

Complaints from disgruntled clients in general—i.e., not just in relation to euthanasia—are a stress factor for veterinarians (24), and a complaint to the VDB can certainly be perceived as a clear complaint. In such cases, it is common for the veterinarian or clinic in question to be negatively portrayed in the media, from the local newspaper to social media, which obviously does not contribute to a good working environment (25, 26). Although a complaint can cause negative stress for the veterinarian, it can be argued that it is important for society that clients have a voice if they believe that their animal has been mistreated. Such a complaint may also help to correct or prevent systematic errors, thereby improving the general level of veterinary care and professionalism.

## Data Availability

The data analysed in this study is subject to the following licenses/restrictions: in accordance with GDPR, no identifiable data on human identities are retained at the University. Non-identifiable data can be shared on request. Requests to access these datasets should be directed to Lotta.Berg@slu.se.
